# Progress in Phototherapy

**Published:** 1903-05-16

**Authors:** 


### PROGRESS IN PHOTOTHERAPY.

Now that the light treatment for dermatoses is becoming more generally trusted and recognised, there have been further attempts at reducing the cumbrous and expensive apparatus first introduced by Finsen. One of these newer forms has been devised by Reyn, a colleague of Finsen's, and termed the "Finsen-Reyn" lamp. By this, only one patient at a time can be treated, which in itself is not a disadvantage, allowing as it does for privacy. The lamp consists of an arc lamp, a concentrating telescope, and a compressor. A current of 20 ampères at 55 volts is required, and the carbons, which are inclined at an angle, are fed automatically. The condenser is only 10 inches long. By specially devised water-cooling arrangements the arc can be brought close to the rock-crystal lenses. In this there are several chambers, two of which contain stationary water (distilled), and two are supplied with running water. Graham,[1] who supplies the description, states that rather more than 15 minutes are required for a sitting, and that the advantages are—lessened cost per patient, apparently greater rapidity in obtaining satisfactory results, that each patient can be treated privately, its mobility, and the greater ease with which the more inaccessible parts of the body are reached.

A lamp burning carbon or iron electrodes is described by Stopford Taylor.[2] It is a small apparatus with a water jacket, and its special features consist in its shape, which allows of its being brought with ease against places difficult of access with other lamps, and the fact that the lamp is fixed to a bracket which is one with a specially-made chair, so that pressure can be made and maintained in any position and direction.

Piffard[3] speaks highly of a lamp constructed after the pattern of Görl with iron electrodes and three spark gaps excited by means of a static machine with Leyden jars as condensers. In this way, using one-eighth inch spark gaps, a light very rich in ultra-violet rays is produced, and a number of separate lamps arranged in series can be actuated by the same current.

O'Brien[4] states that most of the objections urged against the French lamp can be obviated by prolonging the duration of each sitting and increasing the intensity. With it he has found the reactions in all cases better, and penetration to the deeper tissues more manifest.

The usefulness of the light treatment has not yet reached its limit, for Max Heine[5] reports a case of chronic pemphigus of two years' duration, and which had not responded to apparently every known remedy, but which was readily and completely cured by light. The treatment here consisted in the light from an arc lamp of 15 ampères being directed upon the patient's skin. An extraordinary improvement was noticed even after the first sitting. Bullæ began to dry up, and no new lesions formed, and the patient was well after nine sittings in 13 days. Blue light was directed upon two bullæ which had been scratched, and healed them. The author asks whether the fatal foliaceous pemphigus might not yield to some such treatment.

Kime[6] also states confidently that laryngeal tuberculosis may be successfully treated by light; and that after a few applications pain disappears and aphonia diminishes. He has used the external application of light as an adjuvant in the open-air treatment of phthisis, and regards this as supplying the thing needful in patients for whom the latter would have little chance by itself. In his experience, out of 60 cases of pulmonary tuberculosis treated in this way, the disease was arrested in 12, improvement marked in 11, no improvement in 1, 5 being dismissed as incurable, while the remaining 31 are yet under treatment. His method consists in directing an intense blue light from a reflector of 36 inches in diameter and 3 feet focal length upon the patient's chest, a patch 8 inches in diameter being treated at a time for two or three hours a day. The source of light is the sun. He states that after this the lungs are engorged with blood, as the skin is in the treatment for lupus, which shows itself in a transitory dyspnœa, that dulness is lessened and in early cases normal resonance returns.

According to Hyde and Ormsby[7] they have had more satisfactory results with light in lupus erythematosus than with the *x*-rays. The latter they find tend to aggravate the trouble in many cases.

Mount Bleyer[8] finds that the therapeutic power of iron as a drug can be very greatly hastened and enhanced, by simultaneously exposing the body to direct sunlight or electric light, the good effect beginning almost instantaneously. He speaks well of Tropon as showing important therapeutic results after very short time and giving the least digestive disturbances of any preparation of iron.

That some cancers may be found to react favourably to the *x*-rays would seem to be the case from reports of instances to hand, though in the majority of experiences what little good was gained temporarily soon was lost again. It may be that Margaret Cleaves[9] is right in her suggestion that too rapid a treatment may be attended by the too rapid release of infectious material from the tumours into the general system and consequent intoxication with the worst results. This writer gives an exhaustive report upon a case of cauliflower growth of the cervix, stated to be too far gone for surgical intervention which was treated by ultra-violet light and *x*-rays. The treatment consisted in 110 applications in the space of five months and one week. For the first two and a-half months treatment was daily, then three times a week, etc. Light and *x*-rays were used at the same sitting as a rule, though during menstruation the general light bath only was used. As a result there was a gain of 8 lbs. over any weight in her life; all bad odour and hæmorrhages disappeared; the appetite returned with good digestion; sleep was normal and no sense of anything wrong remained. Locally, the uterus was bound down and drawn to the left by scar tissue, but no tumour or secondary growths were to be found. The applications of *x*-rays were intermitted when rigors occurred and the treatment not pushed at all. The author quotes other cases of pelvic cancer, some of which have derived benefit and others not so. She has found light generally applied useful in the treatment of anæmia and tuberculosis, including tubercular ulceration of the larynx. Cleaves is not alone in her report of a successful case of carcinoma cervicis, for Scully[10] records two cases where he believes the disease to be obliterated after use of the *x*-rays. Hæmorrhage and offensive discharges ceased, ulcerations healed, and growths sloughed or shrivelled up. The general condition of patients improved very much. His advice is that the rays should be employed after hysterectomy or other palliative procedure.

A case of alveolar melanotic sarcoma which had recurred after operation was treated by Walker,[11] of Evansville, with the *x*-rays before the wound of the second operation had healed. Improvement was immediate, the wound cicatrized in two weeks, and all indurations had disappeared at the end of three months. The patient seemed entirely cured.

In a case of Coley's,[12] where a sarcoma involved the left femur to the extent of two-thirds of the shaft, the *x*-rays, which were applied on amputation being refused, produced a decrease in size in the tumour of 1 inch in three months, while there was a slight increase in body weight. The applications were made three or four times weekly.

Coley[13] himself only advises the use of the rays in inoperable cases. He has seen 75 malignant tumours treated in this method, 27 being sarcomata and 20 cancers of the breast. Five round-celled sarcomata were entirely cured. In 16 cancers of the breast which were inoperable after recurrence, the recurrent nodules frequently disappeared, but soon reappeared. No case of cancer had remained long enough to be considered absolutely cured. He has treated cases for eight months without the recurrent tumours disappearing.

In Brook's[14] experience two patients the subjects of cancer of the breast had been apparently entirely freed from the malignant growth. In these no operation had been performed. In a third case, where the breast had previously been removed, nodules disappeared and the general condition improved greatly, but the case terminated fatally.

[1] Lancet, Feb. 14, 1903. [2] Lancet, Feb. 21, 1903. [3] Med. Rec., March 7, 1903. [4] Dublin Jour. Med Sci., February 1903. [5] Treatment, September 1902. [6] Med. Rec., Nov. 1, 1902. [7] Med. Rec., Jan. 10, 1903. [8] Med. Rec., Jan. 31, 1903. [9] Med. Rec., Dec. 13, 1902. [10] Brit. Med. Jour., Feb. 28. [11] Med. Rec., Nov. 8. [12] Anns. of Surg., October 1902. [13] Med. Rec., Jan. 10. [14] Brit. Med. Jour., Oct. 25.

*(To be continued.)*

